# Biodegradable Polycarbonate Iongels for Electrophysiology Measurements

**DOI:** 10.3390/polym10090989

**Published:** 2018-09-05

**Authors:** Alexander Y. Yuen, Luca Porcarelli, Robert H. Aguirresarobe, Ana Sanchez-Sanchez, Isabel del Agua, Usein Ismailov, George G. Malliaras, David Mecerreyes, Esma Ismailova, Haritz Sardon

**Affiliations:** 1 POLYMAT, Joxe Mari Korta Center, University of the Basque Country UPV/EHU, Avda. Tolosa 72, 20018 Donostia-San Sebastian, Spain; ayuen08@gmail.com (A.Y.Y.); luca_porcarelli001@ehu.eus (L.P.); roberto.hernandez@ehu.eus (R.H.A.); iisabel.delagua@emse.fr (I.d.A.); david.mecerreyes@ehu.es (D.M.); 2 Electrical Eng. Division, Dept. of Eng., University of Cambridge, 9 JJ Thomson Avenue, Cambridge CB3 0FA, UK; abs50@cam.ac.uk (A.S.-S.); gm603@cam.ac.uk (G.G.M.); 3 Department of Bioelectronics, Ecole Nationale Supérieure des Mines, CMP-EMSE, MOC, 13541 Gardanne, France; usein.ismailov@mines-stetienne.fr (U.I.); ismailova@emse.fr (E.I.); 4 Ikerbasque, Basque Foundation for Science, 48011 Bilbao, Spain

**Keywords:** iongels, polycarbonate, electrophysiology, electrodes, biodegradable

## Abstract

In recent years, gels based on ionic liquids incorporated into polymer matrices, namely iongels, have emerged as long-term contact media for cutaneous electrophysiology. Iongels possess high ionic conductivity and negligible vapor pressure and can be designed on demand. In spite of the extensive efforts devoted to the preparation of biodegradable ionic liquids, the investigations related to the preparation of iongels based on biodegradable polymers remain scarce. In this work, biodegradable polycarbonate-based iongels are prepared by ring-opening polymerization of *N*-substituted eight ring membered cyclic carbonate monomers in the presence of imidazolium lactate ionic liquid. Our iongels are able to take up 10–30 wt % of ionic liquid and become softer materials by increasing the amount of free ionic liquid. Rheological measurements showed that the cross-over point between the storage modulus G′ and loss modulus G″ occurs at lower angular frequencies when the loading of free ionic liquid increases. These gels are able to take up to 30 wt % of the ionic liquid and the ionic conductivity of these gels increased up to 5 × 10^−4^ S·cm^−1^ at 25 °C as the amount of free ionic liquid increased. Additionally, we assess the biodegradation studies of the iongels by immersing them in water. The iongels decrease the impedance with the human skin to levels that are similar to commercial Ag/AgCl electrodes, allowing an accurate physiologic signals recording. The low toxicity and biodegradability of polycarbonate-based iongels make these materials highly attractive for cutaneous electrophysiology applications.

## 1. Introduction

Today, cutaneous electrodes are used to monitor specific physiological functions during routine medical procedures using standard electrodes based on silver/silver chloride (Ag/AgCl) conductive layers that are applied directly on the skin of the patient. The quality of the recording depends on the interface impedance between the skin of the patient and the working electrode. This interface impedance is usually inferior in dry skins. Consequently, aqueous contact media are used to reduce the impedance across the electrode and skin interface. Hydrogels—polymer networks extensively swollen with water—have dominated the field due to their good mechanical properties, high water content and good conductivity. In spite of being excellent candidates for short-term recording, hydrogels present some shortcomings during long-term recording such as water evaporation during recording [1,2]. As water evaporates, the impedance of electrodes increases and the quality of the recording decreases. In this sense, the hydrogels must be frequently replaced or refilled, causing discomfort to the patients [1]. Another important drawback of hydrogels is related to the requirements of an adhesion promoter of Ag/AgCl electrodes to ensure good contact with the skin. Adhesion promoters may leave residues on the patient or cause irritation and allergic reaction [3,4,5]. 

Hence, the development of stable cutaneous electrodes that do not require aqueous contact media or a promoter has been attracting the attention of long-term electrophysiology. Dry electrodes that do not require contact media were proposed as an alternative [6,7,8]. However, the lack of an electrolyte means that artifacts caused by the movement of the patient are more likely to occur during recording [9]. Recently, gels based on Ionic Liquids (ILs) incorporated into polymer matrices—namely iongels or ionogels—were suggested as alternative contact media in the field of bioelectronics, including long-term cutaneous electrophysiology [10,11]. ILs are organic salts with melting points near room temperature. ILs have been widely discussed as sustainable solvents due to their unique chemical properties, including intrinsically high ionic conductivity and negligible vapor pressure, which may overcome the evaporation limitations of aqueous contact media [12,13,14]. Leleux et al. proposed an iongel based on 1-ethyl-3-methylimidazolium ethyl sulfate ionic liquid embedded within a poly(ethylene glycol) diacrylate network with impedance values competitive to commercially available hydrogels that allowed longer recordings due to negligible evaporation [15]. Besides the excellent results achieved with this first generation of iongels, the employed ionic liquid was not biocompatible. In order to fight this issue, our group recently reported the use of biocompatible, biodegradable and low toxicity iongels based on a cholinium lactate ionic liquid for accurate electrocardiography measurements [16]. Although the used ionic liquid was biodegradable and biocompatible, the main drawback of these iongels was related to the negligible biodegradability of the utilized polymer network that was based on chemically inert carbon chain backbones. This fact limits the implementation of these systems, especially as biodegradable implantable electrodes. In addition, we envisioned that iongel residues left on the skin of the patient could be washed away faster in the case of hydrolysable backbones. In our search for a biodegradable backbone, we have found that polycarbonates offer desirable mechanical properties, biocompatibility, and fast biodegradation kinetics in water [17]. 

Recently, Sardon and coworkers explored the use of *N*-substituted eight membered cyclic carbonate monomers to obtain polycarbonates intended for the biomedical field [18,19]. In this work, we prepared polycarbonate matrices by direct polymerization of cyclic carbonates in the presence of bifunctional cyclic carbonates and PEG-diol. Importantly, the obtained hydrogels were not only fully biodegradable but their degradation products were also non-toxic and showed great potential in the area of the wound dressing. Taking advantage of the fact that this material is biodegradable, in this paper, we combined our polycarbonate matrices with biocompatible ILs to prepare innovative contact media for cutaneous electrophysiology. We envision a good compatibility of the polymer backbone with the ionic liquid due to the presence of tertiary amines in the main polycarbonate backbone. The biodegradability, rheological, and electrical properties of the gels were studied. Finally, the iongels were mounted onto PEDOT:PSS based electrodes and used in electrocardiography (ECG) recordings. 

## 2. Materials and Methods

Lactic acid solution (≥85%), 1-butyl-3-methylimidazolium chloride (≥99%), ethylene glycol (≥99%), polyethylene glycol 8000 (USP), 4-dodecylbenzenesulfonic acid (≥95%), acetic acid (≥99%), 3-methacryloxypropyltrimethoxysilane (>98%), and 1,8-diazabicyclo[5.4.0]undec-7-ene (≥99%) were purchased from Sigma Aldrich (St. Louis, MO, USA). Clevios^TM^ PH-1000 (PEDOT:PSS) was purchased from Heraeus (Hanau, Germany). Amberlyst^TM^ A-26 (OH-form) and analytical grade solvents were purchased from Fisher Scientific (Hampton, NH, USA). Deuterated solvents were purchased from Deutero (Kastellaun, Germany). The *N*-substituted eight membered cyclic carbonates: 6-methyl-1,3,6-dioxazocan-2-one (8MC) and 6,6′-(ethane-1,2-diyl)bis(1,3,6-dioxazocan-2-one) bis-8MC were synthesized using the previously reported procedures [19,20]. ILs were prepared using Amberlyst^TM^ A-26 (OH-form) exchange resin following the procedure in the literature [21], ILs were stored inside a N_2_ filled glovebox (H_2_O and O_2_ < 5 ppm). ^1^H- and ^13^C-NMR spectra were recorded with a Bruker Avance DPX 300 spectrometer. The NMR chemical shifts were reported as δ in parts per million (ppm) relative to the traces of non-deuterated solvent. Fourier transform infrared-attenuated total reflection (FTIR-ATR) spectroscopy was performed with a Bruker Alpha. 

### 2.1. Preparation of Polycarbonate Iongels

A 12 mL vial was charged with 8MC (0.310 g, 2.2 mmol), bis-8MC (0.120 g, 0.42 mmol), PEG_8000_ end capped diol (0.030 g), and a known amount of ionic liquid. Then, DBU (0.036 g) in 2 mL of DCM was added to the solution and the vial was sealed. Then, the vial was placed into an oil bath at 40 °C for 24 h. Then, the vial was taken out of the oil bath and placed on a flat surface. The cap was then removed for the DCM to evaporate slowly at room temperature, resulting in the formation of the iongel. Before measuring the ionic conductivity of the iongels, they were carefully dried in a conventional oven at 50 °C (12 h) and later a Buchi^®^ (Meierseggstrasse, Switzerland) oven at 50 °C (24 h) under vacuum.

### 2.2. Characterization of Iongels

Rheology measurements on the iongels were conducted on an Anton PaarPhysica MCR 101 rheometer (TA instruments, Lukens Drive New Castle, DE, USA) using oscillatory tests with parallel plate geometry. Angular frequency sweeps from 0.0628 s^−1^ to 314 s^−1^ at constant strain amplitude (γ = 1%) were applied at 25 °C. Later, G′ and G″ values were plotted versus frequency. Biodegradability assessments of iongels were performed using a glass bottle filled with 250 mL of MilliQ water. Next, the polycarbonate iongel was placed flat at the bottom-center of the bottle. The bottle was then sealed and kept at room temperature. Once the iongel had degraded or was no longer visible, the water was removed using a rotary evaporator and the remaining residues were then characterized by ^1^H- and ^13^C-NMR and FTIR-ATR.

### 2.3. Device Fabrication

The electrodes were fabricated using a previously published method [15]. In short, the plastic polyimide (Kapton HN, Dupont, Delaware, DL, USA) electrodes were laser-cut to a thickness of 125 mm and an active area of 0.5 cm^2^. Then, 10 nm of chromium and 100 nm of gold were thermally evaporated onto the electrodes. A solution of PEDOT:PSS, ethylene glycol, 4-dodecylbenzenesulfonic acid, and 3-methacryloxypropyltrimethoxysilane (weight ratio of 80/10/0.4/1) was then drop casted (5 µL) onto the active area of the electrode. The PEDOT:PSS film was then baked at 110 °C. Later, the IG-30 was then deposited on top of the PEDOT:PSS layer and left overnight at room temperature and in ambient conditions. To finish the device fabrication, the interconnection side of the electrode was attached to a snap button, and a protective dielectric paint was applied for the insulation of the device.

### 2.4. Impedance Measurements and Physiological Data Acquisition

The skin impedance measurements were acquired using a potentiostat (Autolab equipped with FRA module, Metrohm B.V., Herisau, Switzerland), using a three electrodes configuration [22]. The reference electrode was placed on the elbow. The working and counter electrodes were placed 2 cm apart on the forearm. The reference and counter electrodes were Ag/AgCl electrodes (Ambu Blue Sensor N, N-00-S/25, Ballerup, Denmark). 0.95 cm diameter contact area) and the working electrode (WE) was the one under study [16]. Impedance was measured within a range of frequencies (10^−1^–10^5^ Hz) and the measurements were repeated two times. Electrocardiography (ECG) recordings were performed on volunteers. An RHD2216 amplifier chip from Intan Technologies (Los Angeles, CA, USA) was used to record the ECG signals. The signals were sampled at 1.1 kHz at 16 bits. A first-order high pass analog filter at 0.1 Hz and a third-order low pass analog filter at 100 Hz filters were used. ECG recordings were performed using three electrodes. The ground electrode was placed on the right leg and two electrodes on each wrist of a healthy volunteer. The reference electrode was the Ag/AgCl electrode (Ambu Blue Sensor N, N-00-S/25, 0.95 cm diameter contact area) and two test electrodes were made of PEDOT:PSS covered by IG-30. All measurements were carried out three times while the volunteer was not moving [3,6,23]. All volunteers provided informed signed consent to participate in the study.

## 3. Results and Discussions

### 3.1. Preparation of the Iongels

**Scheme 1:**
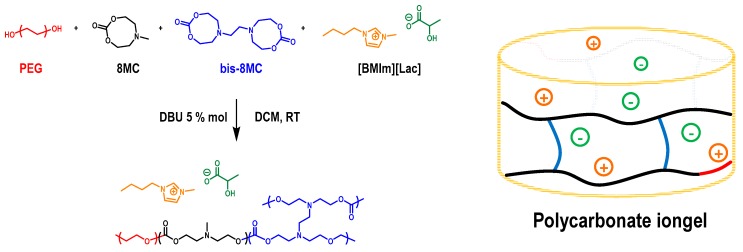
In-situ preparation of iongels via one-step ring-opening polymerization of 6-methyl-1,3,6-dioxazocan-2-one (8MC) and 6,6′-(ethane-1,2-diyl)bis(1,3,6-dioxazocan-2-one) bis-8MC initiated from polyethylene glycol (PEG), in the presence of an organic catalyst (DBU) and the ionic liquid [BMIm] [Lac].

Polycarbonates based on *N*-substituted cyclic carbonates are receiving growing attention in recent years for their potential applications to biological and medical engineering [18,19,24]. In this work, we prepared polycarbonate-based iongels via one-step ring-opening polymerization, as illustrated in Scheme 1. Ring opening polymerization allows for the synthesis of a polycarbonate matrix that can be degraded via hydrolysis of the carbonate linkages. The ring opening polymerization of monofunctional and bifunctional cyclic carbonates was initiated from the hydroxyl end group of polyethylene glycol initiators, in the presence of an organic catalyst, DBU. DBU was chosen as a catalyst as it has been demonstrated to be effective in the synthesis of polycarbonates from *N*-substituted cyclic carbonates. 

**Figure 1:**
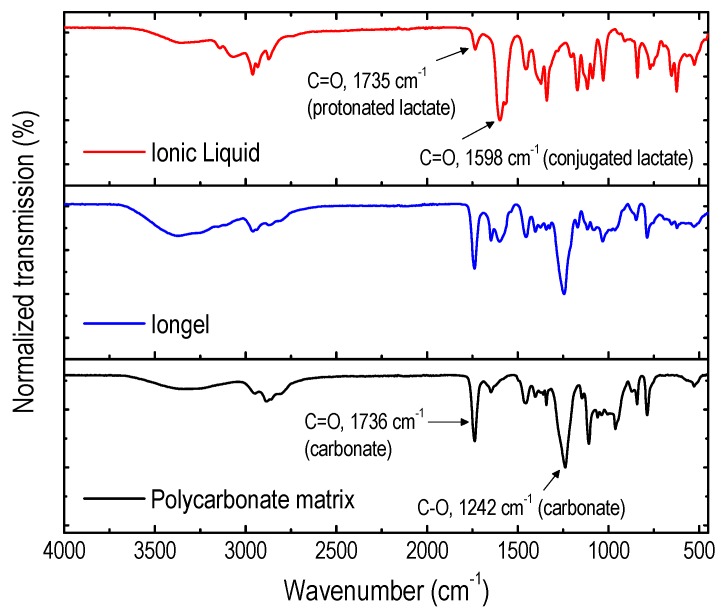
The FTIR-ATR characterization of the ionic liquid [BMIm] [Lac], the polycarbonate iongel with 30 wt % [BMIm] [Lac] (IG-30), and the polycarbonate matrix without any ionic liquids.

Our design is based on the ability of bifunctional cyclic carbonates to create crosslinking points between neighboring polymer backbones, thus forming a three-dimensional polymer network. Iongels were synthesized using a molar ratio of PEG:DBU:cyclic carbonate monomers at 1:5:100 in DCM (monomers concentration: 1 M). The ratio between the monofunctional and bifunctional carbonate was chosen to 80:20 mol% based on our previous results to obtain gels with appropriate mechanical properties and swelling behaviors. We performed the polymerization in the presence of different ionic liquids and different ionic liquid concentrations (0 to 35 wt %). In order to facilitate the mixing of all components, a little amount of dichloromethane was used prior to the polymerization. Using this one pot synthesis approach, the ionic liquid will be trapped inside the polymer network while the volatile solvent can be removed by simple evaporation. During our first attempt to prepare iongels, we selected the ionic liquid Cholinium Lactate, [Ch] [Lac], because of its biocompatibility, biodegradability and low toxicity [16,25,26]. A range of 10 to 30 wt % of [Ch] [Lac] was added to the starting solution. However, none of these formulations produced self-standing iongel films and only an increase in viscosity was observed. We hypothesize that the primary alcohol group in the cholinium cation acted as an initiator of the ring opening polymerization process. An excess of initiators can yield to short polycarbonate chains with an insufficient degree of crosslinking to form iongels with good mechanical properties [19]. For the sake of comparison, in our previous work, we prepared iongels containing up to 60 wt % [Ch] [Lac] using free radical polymerization techniques, which are less sensitive to the presence of functional groups [27]. Looking for alternative ionic liquids to [Ch] [Lac], we selected 1-butyl-3-methylimidazolium lactate [BMIm] [Lac]. The toxicity of imidazolium-based ILs is largely determined by the length of the alkyl chain. For this reason, we chose an imidazolium-based IL with a short butyl chain. We chose the intrinsically biocompatible lactate anion as the counter ion of the ionic liquid. We were able to consistently prepare iongels up to 35 wt % [BMIm] [Lac], however, the gels with the higher IL content were extremely delicate and difficult to handle. The iongels were coded as imidazolium IG-0, IG-10, IG-20, and IG-30 where the number represents the weight percent of the free ionic liquid. The polymerization was confirmed by detailed spectroscopic characterization of the iongels using FTIR-ATR (Figure 1). The spectrum of IG-30 shows two bands at 1736 and 1248 cm^−1^ from the C=O and C–O stretching vibrations of the polycarbonate structure, according to the literature [19]. The same bands are clearly observable in the spectrum of an IL-free polycarbonate matrix, measured as a reference. In addition, we observed a band at 1598 cm^−1^ attributed to the C=O stretching of the lactate anion. In summary, we can observe characteristic [BMIm] [Lac] and polycarbonate signals within our IG-30 samples, the indicating successful polymerization of the cyclic monomers in the presence of [BMIm] [Lac]. 

Notably, the alcohol group in the lactate anion did not affect the polymerization, as the secondary alcohol of the lactate group is less reactive as an initiator for the ring opening polymerization because of the steric hindrance.

### 3.2. Characterization of the Iongels

**Figure 2:**
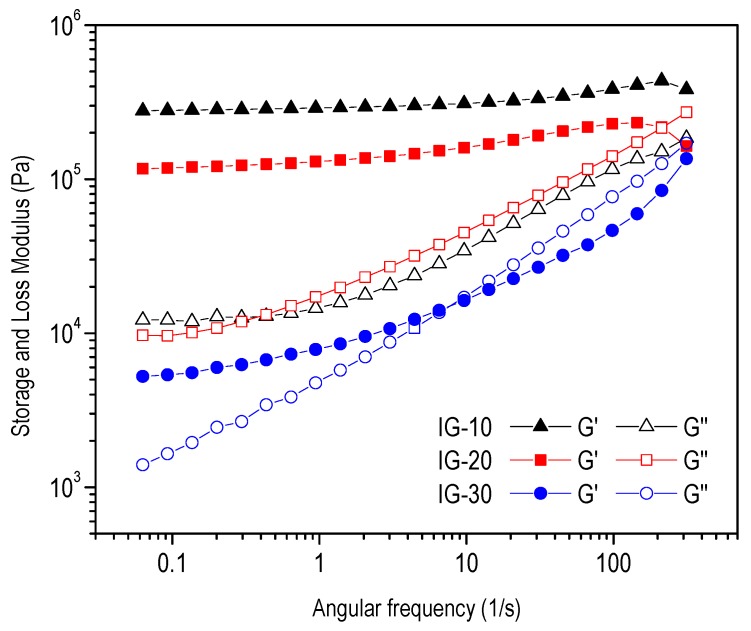
The rheological studies using oscillatory tests were performed for the polycarbonate iongels with 10 wt % [BMIm] [Lac] (IG-10), 20 wt % [BMIm] [Lac] (IG-20), and 30 wt % [BMIm] [Lac] (IG-30). Elastic moduli (G′) are marked with solid symbols and loss moduli (G″) are marked with open symbols.

In order to study the physicochemical properties of the gels, rheological measurements were performed for a series of polycarbonate iongels, loaded with different amounts of [BMIm] [Lac] (10–30 wt %). The mechanical properties of iongels are important properties because as the iongel has to be in contact with the skin, the interface has to be soft and flexible to absorb mechanical shocks. Therefore, the rheological behavior of the cross-linked materials was investigated by measuring the elastic (G′) and the viscous (G″) moduli as a function of the frequency at room temperature (Figure 2). For IG-10, the iongel with the least amount of ionic liquid (10 wt %), we observed G′ dominating G″ for the entire range of angular frequencies tested suggesting that the material remained chemically cross-linked. Interestingly, as we increased the ionic liquid content from 10 to 20 wt %, we can see that IG -20 behaves as an elastic gel for almost the entirety of the range of frequencies. At the angular frequency of 213 s^−1^, there is a crossover point between G′ and G″ in which the material ceases to behave as a gel and begins to behave as a viscous liquid. As we arrive to the 30 wt % IG-30, we see this cross-over point shift further to lower angular frequencies, from 213 to ~6 s^−1^. It should be noted, however, that the cross-over point at 6 s^−1^ is not as obvious at the one at 213 s^−1^. Although IG-30 behaves as a viscous material at higher angular frequencies (>10 s^−1^), the gel-like behavior at lower angular frequencies (<10 s^−1^) is more than sufficient for our application. Our intended applications of these materials are for electrodes to be placed on areas of the body with much less movement. As expected, the introduction of the free ionic liquid into the polymer network acts as a plasticizer and reduces the mechanical strength of the iongels as revealed by the decreased G′ and G″ curves of IG-30 in comparison to the IG-10. This indicated that the gels were softer as we increased the ionic liquid content, and this is indeed what we observed while handling the materials.

### 3.3. Ionic Conductivity Measurements

Due to the presence of the free IL embedded in the polymer matrix, iongels in most of the cases present higher ionic conductivities than other solid polymer electrolytes which is an important parameter for electrophysiology applications. Thus, we used electrochemical impedance spectroscopy to measure the ionic conductivity (σ) of the iongels, the results are shown in Figure S1. As expected for the sample IG-10, we obtained ionic conductivity values that were low between 8 × 10^−8^ at 5 °C and 1.7 × 10^−6^ S·cm^−1^ at 40 °C. The conductivity values were gradually increasing with the increasing free ionic liquid amount in the iongel. Thus, the ionic conductivities increased up to 3.5 × 10^−7^ at 5 °C and 1 × 10^−5^ S·cm^−1^ at 40 °C for IG-30 with 30 wt % of ionic liquid. 

**Figure 3:**
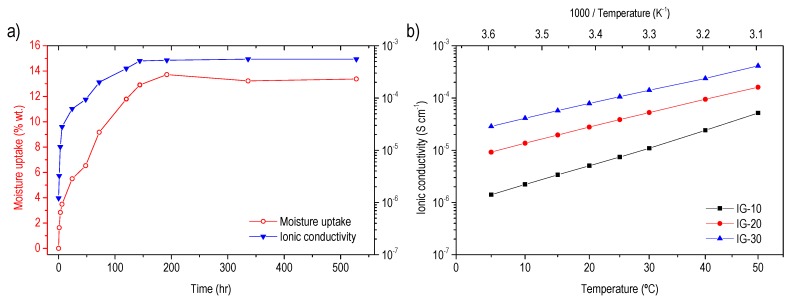
The moisture uptake and ionic conductivity at 25 °C of a polycarbonate iongel with 30 wt % [BMIm] [Lac] (IG-30) stored inside a humidity controlled environmental room for a week (**a**); ionic conductivity of polycarbonate iongels with 10 wt % [BMIm] [Lac] (IG-10), 20 wt % [BMIm] [Lac] (IG-20), and 30 wt % [BMIm] [Lac] (IG-30) stored inside a humidity controlled environmental room (RH 55%) (**b**).

Besides the relatively low ionic conductivity of the synthesized gels for electrophysiology, when the samples were exposed to open air, they started to absorb moisture (Figure 3a). After 24 h, IG-30 absorbed 11 wt % of water and its room ionic conductivity increased from 1.2 × 10^−7^ to 6.5 × 10^−5^ S·cm^−1^ (two orders of magnitudes). It is known that moisture can act as a strong dopant for ionic conductivity, consequently, we investigated the effect of moisture uptake on the ionic conductivity. We exposed the samples to the atmosphere of a humidity controlled environmental room over a period of several days (relative humidity, RH 55%). Over the first six days, the amount of water adsorbed in the gel quickly rose up to 14 wt % The ionic conductivity followed the same trends and a maximum conductivity corresponding to ~5 × 10^−4^ S·cm^−1^ was measured. After six days, the amount of water absorbed from the atmosphere did not change significantly, and the ionic conductivity reached a plateau. All samples were subjected to similar testing and exhibited an increase of ionic conductivities by one or two orders of magnitude (Figure 3b). Despite the initially measured values of ionic conductivity being quite unsatisfactory, the values obtained from the gels exposed to environmental humidity were comparable to the one previously reported by Isik et al. [16]. Previous studies showed that the accumulation of perspiration under the electrode can decrease the impedance of the electrode [9]. We assume that our iongels can perform similarly thanks to their water adsorption capacity.

### 3.4. Hydrolytic Degradation

**Figure 4:**
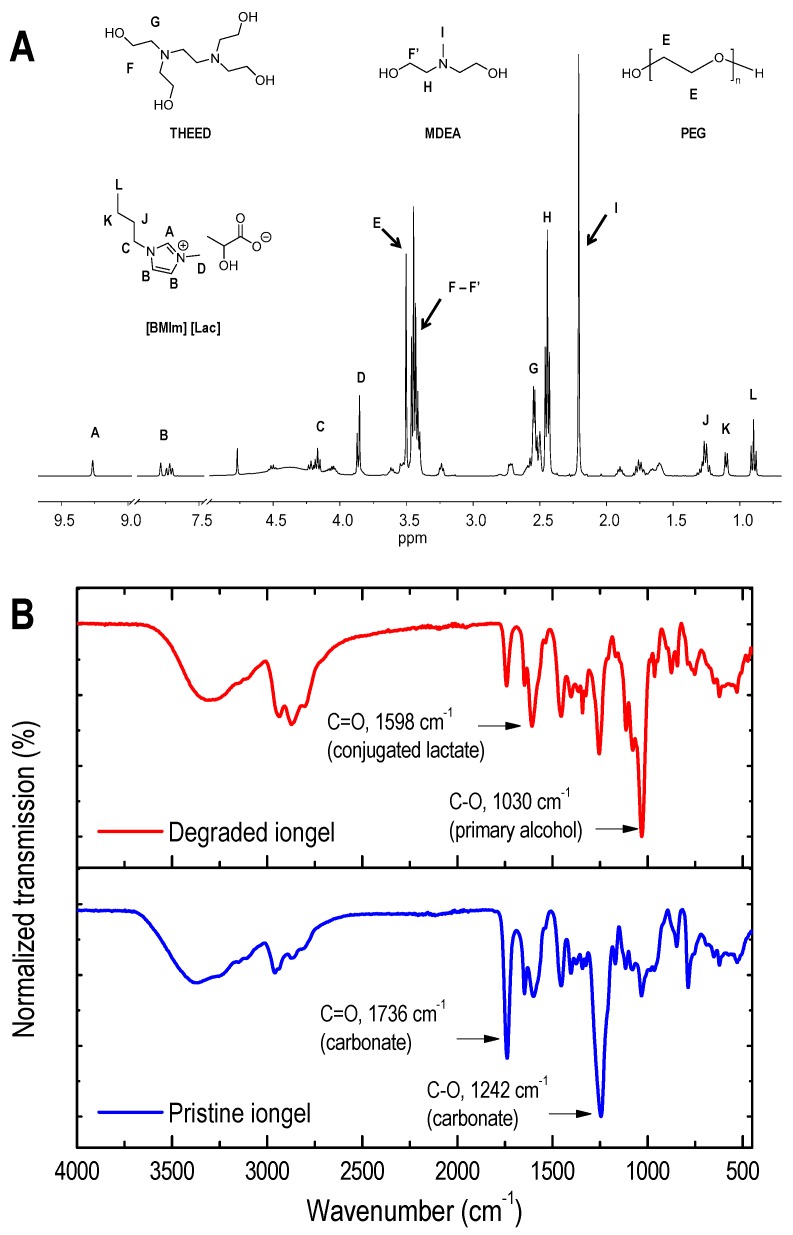
The hydrolysis products of a polycarbonate iongel with 30 wt % [BMIm] [Lac] (IG-30) were characterized by ^1^H-NMR in d_6_-DMSO (**A**) and FTIR-ATR (**B**). The iongels have degraded into their starting materials: *N*,*N*,*N′*,*N′*-tetrakis(2-hydroxylethyl)ethylenediamine (THEED), *N*-methyldiethanolamine (MDEA), polyethylene glycol (PEG), and [BMIm] [Lac].

A key advantage of polycarbonate backbones in comparison to carbon chain backbones is the ability to hydrolyze them in the presence of water. In our previous work, we demonstrated the hydrolytic degradation of polycarbonates matrixes derived from *N*-substituted 8-membered cyclic carbonates to non-toxic byproducts [19]. Based on our previous findings, we envision good degradation properties in water for our newly synthesized iongels. We evaluated the degradation process of IG-30 by submerging the samples in deionized water at room temperature. Within minutes, the iongel started to lose its three-dimensional structure. After two hours in water, the iongel appeared to be partially degraded, whereas after 5 h IG-30 was completely degraded (Video S1). With respect to our previous report, the degradation of the polycarbonate matrixes loaded with ionic liquid was significantly faster [19]. We characterized the products of hydrolytic degradation of the iongels by ^1^H-NMR and FTIR-ATR spectroscopy. Both techniques provide evidence for the degradation of polycarbonates back into its starting materials: *N*,*N*,*N′*,*N′*-tetrakis(2-hydroxylethyl)ethylenediamine, *N*-methyldiethanolamine, polyethylene glycol, and [BMIm] [Lac]. The absence of the typical methylene signals neighboring the carbonate group at 4.26 ppm and 2.77 ppm suggested the degradation of the polycarbonate structure. The degradation of the polycarbonate structure was further evidenced by the absence of the carbonyl signal by ^13^C-NMR spectroscopy (Figure S2). The analysis of the decomposition products evidence for the presence of the starting materials of the 8-member cyclic carbonate monomers. We observed a multiplet at 3.42–3.40 ppm, a triplet at 2.55 ppm and a singlet at 2.21 ppm corresponding to the protons of *N*-methyldiethanolamine, the starting material of 8MC. We also observed two multiplets at 3.46–3.43 and 2.55–2.54 ppm corresponding to the protons of *N*,*N*,*N′*,*N′*-tetrakis(2-hydroxyethyl) ethylenediamine, the starting material of bis-8MC. The signals of the PEG appeared at 3.50 ppm. Along the decomposition products of the polycarbonate matrix, we observed the typical imidazolium signals of [BMIm] [Lac] at 9.27, 7.79–7.72, 4.17, 3.87–3.85 and 1.27–0.90 ppm. As expected, the cation of the ionic liquid was stable in water and did not degrade. We also carried out the characterization of the residues with FTIR-ATR (Figure 4B). The intensity of the carbonate stretch at 1740 cm^−1^ decreased, supporting our hypothesis of a successful degradation of the carbonate linkages. At the same time, a new C–O stretching band at 1030 cm^−1^, which was previously absent in the pristine iongel, appeared. We attributed this band to the primary alcohol produced by the hydrolysis of the carbonate group. In addition, we were able to identify [BMIm] [Lac] amongst the residues by its characteristic C=O stretching band at 1598 cm^−1^.

### 3.5. Electrode Fabrication and Testing

**Figure 5:**
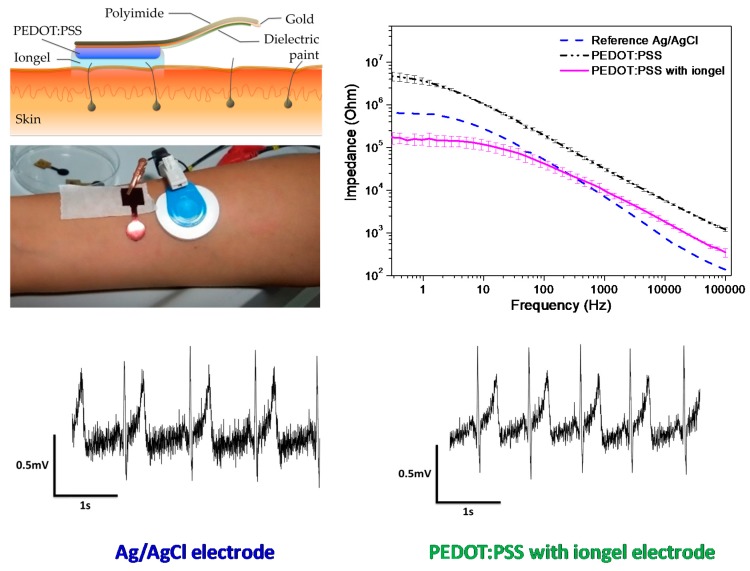
An exploded view of the electrode and all of the different materials used along with a photograph of the electrode (**top left**). Comparison of the impedance on skin recordings with different working electrodes at a frequency range between 0.1 and 100,000 Hz (**top right**). Medical electrode (blue), PEDOT:PSS electrodes (black), and PEDOT:PSS electrodes with a polycarbonate iongel having 30 wt % [BMIm] [Lac] (pink). Electrocardiogram recordings were taken from the wrists of the volunteer (**left center**). The standard medical Ag/AgCl electrode and the IG-30 electrode were evaluated (**bottom**).

In order to improve the contact between skin and the electrodes, polycarbonate iongels loaded with 30 wt % [BMIm] [Lac] (IG-30) were incorporated onto electrodes made of gold and PEDOT:PSS conducting polymer (Figure 5 (Top left)). The electrodes were made out of a thin polyimide film that allowed the electrodes to be flexible and conformable to the skin. Impedance measurements of the electrode/skin interface were carried using a three-electrode configuration on a healthy volunteer. The working and counter electrodes were placed on the subject’s forearm, and the reference electrode was placed on the elbow. For this study, commercially available Ag/AgCl electrodes were used for both the counter and reference electrodes. Moreover, our electrodes were used as the working electrodes. To compare our electrodes to the standard medical ones, an additional impedance measurement was made with an Ag/AgCl working electrode.

In Figure 5 (Top right), we observed a clear difference in impedance when the polycarbonate iongel was present (pink) or absent (black) from the electrode. When the iongel was incorporated, we saw a decrease in impedance by approximately one order of magnitude across the entire range of frequencies tested. This was particularly favorable because lower impedances generally allow for better signal to noise ratios for cutaneous electrophysiological (ECG) recordings [28]. Impedance measurements were also taken using standard medical electrodes (Ag/AgCl electrode, blue). Our electrodes displayed lower impedances at frequencies below ~100 Hz, which are the frequencies where ECG electrodes generally operate (1–100 Hz). To further confirm the potential of the iongel, ECG measurements were taken from a healthy volunteer using both the IG-30 electrode and the standard medical electrode (Figure 5 (Left center)). The recording performance of the IG-30 electrode was comparable with the standard medical electrode, and the QT intervals which are used to identify numerous heart conditions, such as arrhythmias or hypertrophy could be obtained for both materials. Recordings of approximately 10 min were taken for each material, and the volunteer reported no redness or irritation of the skin. 

## 4. Conclusions

A new family of iongels based on biodegradable polycarbonates and biocompatible ionic liquids were prepared. We propose the use of our iongels as an alternative to conventional aqueous contact media for electrophysiological recordings. By means of FTIR-ATR and rheological measurements, we demonstrate the successful ring opening polymerization of the cyclic carbonate monomers in the presence of the biocompatible ILs. We tested different concentrations of ionic liquids and we found that iongels consisting of 30 wt % [BMIm] [Lac] (IG-30) were the most promising in terms of mechanical properties and ionic conductivity for electrophysiology. We found that our iongels are moderately hygroscopic, and that the water absorbed by the iongels leads to important gains in ionic conductivities. In addition, these iongels showed fast degradability in water at room temperature which can be an interesting feature for implantable electrodes. Impedance measurements showed that our iongels performed better than standard medical Ag/AgCl electrodes at low frequencies. Lastly, we recorded electrocardiography signals of a healthy patient that are comparable to that of a standard medical electrode without causing any irritation to the patient. This work paves the way for the development of bio- and eco-friendly alternatives to aqueous contact media convenient to use in ECG based diagnostics.

## Supplementary Materials

The following are available online at http://www.mdpi.com/2073-4360/10/9/989/s1, Figure S1: Ionic conductivity of as synthetized polycarbonate iongels loaded with ionic liquids measured across a range of temperatures, Figure S2: Residues from the biodegradability testing of IG-30 characterized by ^13^C-NMR in d6-DMSO., Video S1: Timelapse of a polycarbonate iongel degradation in Milli-Q water.
