# Supplementary material for: Biodegradable Polycarbonate Iongels for Electrophysiology Measurements
Source: Polymers (Basel). 2018 Sep 5;10(9):989. doi: 10.3390/polym10090989 (PMC6404095; doi:10.3390/polym10090989)
Supplement: Supplementary file 1 [file polymers-10-00989-s001.pdf]

polymers

Supporting information

Biodegradable Polycarbonate Iongels for Electrophysiology Measurements

Alexander Y. Yuen [1,†], Luca Porcarelli [1,†], Robert H. Aguirresarobe [1], Ana Sanchez-Sanchez [2], Isabel del Agua [1,3], Usein Ismailov [3], George G. Malliaras [2], David Mecerreyes [1,4], Esma Ismailova[3] and Haritz Sardon [1*]

[1] POLYMAT, University of the Basque Country UPV/EHU, Joxe Mari Korta Center, Avda. Tolosa 72, 20018 Donostia-San Sebastian, Spain
[2] University of Cambridge, Dept. of Eng., Electrical Eng. Division, 9 JJ Thomson Avenue, Cambridge, CB3 0FA, UK
[3] Department of Bioelectronics, Ecole Nationale Supérieure des Mines, CMP-EMSE, MOC, 13541 Gardanne, France
[4] Ikerbasque, Basque Foundation for Science, E-48011 Bilbao, Spain
* Correspondence: haritz.sardon@ehu.eus
† These authors contributed equally to this work.

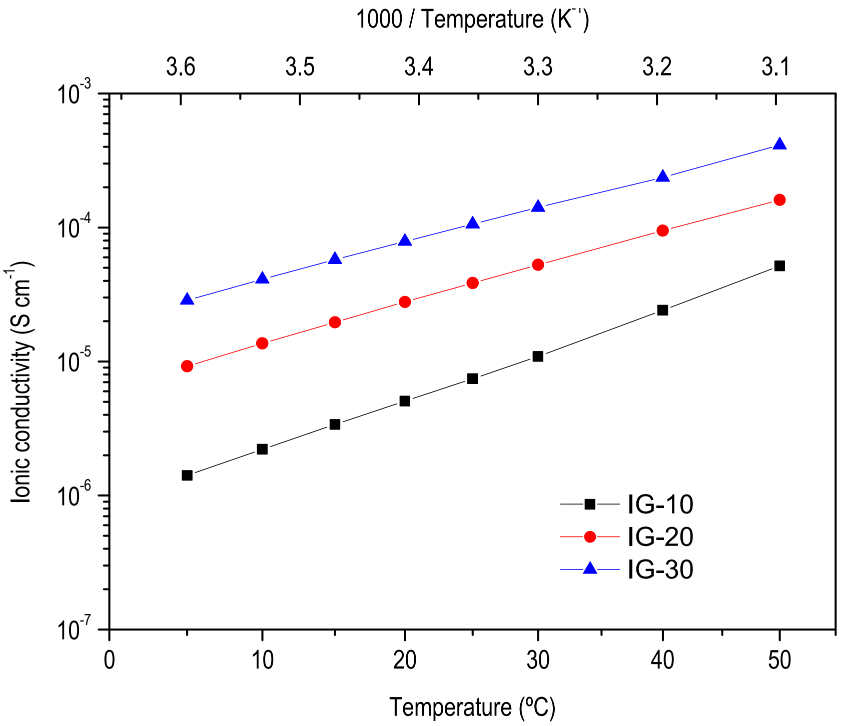

Figure S1. Ionic conductivity of as synthetized polycarbonate ion-gels loaded with ionic liquids measured across a range of temperatures.

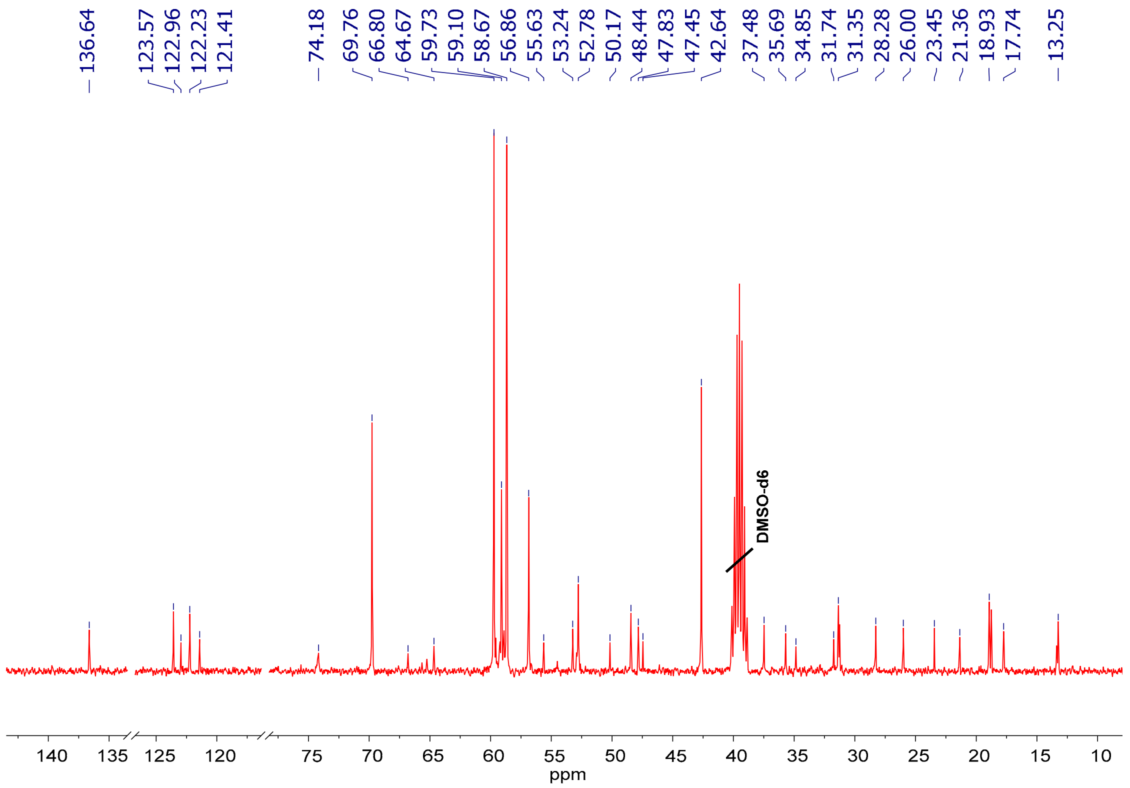

Figure S2. Residues from the biodegradability testing of IG -30 were characterized with ^{13}C NMR in d6-DMSO.

© 2018 by the authors. Licensee MDPI, Basel, Switzerland. This article is an open access article distributed under the terms and conditions of the Creative Commons Attribution (CC BY) license (http://creativecommons.org/licenses/by/4.0/).
